# MiRNAs Predict the Prognosis of Patients with Triple Negative Breast Cancer: A Meta-Analysis

**DOI:** 10.1371/journal.pone.0170088

**Published:** 2017-01-13

**Authors:** Yanli Liu, Yuchao Zhang, Qingfu Li, Junfang Li, Xiaotian Ma, Jinfang Xing, Shouhua Rong, Zhong Wu, Yuan Tian, Jing Li, Liting Jia

**Affiliations:** 1 Department of Reproductive Center, the Third Affiliated Hospital of Zhengzhou University, Zhengzhou, People’s Republic of China; 2 Department of Clinical Laboratory, the Third Affiliated Hospital of Zhengzhou University, Zhengzhou, People’s Republic of China; 3 Department of Stomatology, the People's Hospital of Henan Province, Zhengzhou, People’s Republic of China; 4 Department of Clinical Laboratory, Women and Infants Hospital of Zhengzhou Affiliated to Henan University, Zhengzhou, People’s Republic of China; University of North Carolina at Chapel Hill School of Medicine, UNITED STATES

## Abstract

**Purpose:**

miRNAs are stable and can be extracted from tissues, blood and other body fluid without degradation. miRNAs are abnormally expressed in the presence of a pathological status, including cancer. Therefore, miRNAs are ideal biomarkers for cancer diagnosis and prognosis. Patients with triple negative breast cancer (TNBC) suffer the worst prognosis, although great efforts have been made. Many studies have investigated the role of miRNAs in predicting the outcomes of TNBC patients for better adjustment of treatment. However, results were inconsistent. Thus, we performed a meta-analysis to summarize the published studies for conclusive results.

**Methods:**

Eligible studies from different database were retrieved from the online databases, and we used STSTA 12.0 to analysis the prognostic role of miRNAs in triple negative breast cancer.

**Results:**

Overall high miRNA expression indicated a worse survival with HR value of 1.78 (95% CI: 0.97–3.25). However, subtotal HRs of oncogenic miRNAs and tumor suppressive miRNAs were 2.73 (95% CI: 2.08–3.57; *P*<0.001) and 0.44 (95% CI: 0.21–0.90; *P* = 0.024), respectively, and no heterogeneity was observed within the subgroups.

**Conclusions:**

The miRNAs showed a slightly stronger prognostic value for disease-free survival, relapse-free survival and distant metastasis-free survival compared to the overall survival of TNBC patients. Circulating miRNAs could serve as potential biomarkers for the prognosis of TNBC patients and need further investigation.

## Introduction

Triple negative breast cancer (TNBC) is an important component of heterogeneous breast cancer, and, according to the new refinement, it can be subdivided into four subgroups [1,2]. Because TNBC lacks the expressions of estrogen receptor (ER), progesterone receptor (PgR) and human epidermal growth factor receptor-2 (HER2), few effective treatments, with the exception of conventional surgery, radiotherapy and chemotherapy, can provide benefit for TNBC patients. Tremendous efforts regarding the treatment, such as tailoring adjuvant chemotherapy regimens, the discovery of emerging drugable pathways and the resensitization to radiotherapy or chemotherapy, have been carried out to improve the overall outcome of TNBC patients. However, TNBC has the worst prognosis among all breast cancer subtypes [3–5]. It’s believed that finding appropriate prognostic biomarkers for TNBC patients would allow an optimized treatment selection of regimens that could eventually benefit the patients.

MicroRNAs (miRNAs) are conserved non-coding RNAs (22–25 nt in length), which negatively regulate messenger RNA (mRNA) expression by partially or completely binding to the 3’ untranslated regions (3’UTRs) of the target mRNA [6]. miRNAs have been shown to mediate the cell fate of TNBC by regulating diverse biological processes, such as cell survival, cell cycle arrest, and differentiation[7–10]. Recent studies have confirmed that miRNAs are differentially expressed in tissues, blood and urine of cancer patients and healthy individuals, and they are stable in the presence of severe conditions [11]. Therefore, miRNAs are endowed with the characteristics of ideal biomarkers, and some studies have shown that miRNAs correlate with poor cancer prognosis, including TNBC [12–20]. However, the opposite results were obtained in several other studies [21–25]. Moreover, with regards to patients with TNBC, many factors, such as the race and age of the patients, the selected miRNA(s), the methodology for miRNA detection and the sample source among different studies, varied, leading to inconsistent outcomes [3,26]. Therefore, the aim of this meta-analysis was to systematically study the related references and yield a convincing outcome on whether miRNAs are ideal prognostic biomarkers for patients with TNBC.

## Materials and Methods

### Literature retrieval strategy

Meta-analysis of Observational Studies in Epidemiology group (MOOSE) was followed to guide the performance of this meta-analysis [27]. Two reviewers were assigned to independently retrieve literature from the online databases Pubmed, EMBASE, Web of Science and Chinese National Knowledge Infrastructure (CNKI). The time interval was between January 1st, 1993 and January 1st, 2016. The key words for the literature retrieval strategy included “microRNA”, “miRNA”, “Triple negative breast cancer”, “TNBC”, “basal-like breast cancer”, and “BLBC”. References from eligible publications in the literature were also manually screened for further potential literature.

### Criteria for inclusion and exclusion

Eligible literature met the following criteria: (1) clear identification of patients with TNBC; (2) analysis of miRNA expression in tissues, blood or other body fluids; and (3) investigation of the association between miRNA expression levels and outcome of the TNBC patients. Publications were excluded if they had one or more of the following criteria: (1) focus on all types of breast cancer instead of TNBC only; (2) human tissues were not used; (3) absence of survival outcomes, such as the hazard ratio (HR), 95% confidence interval (CI), and *P* values or insufficient data to calculate the HR and 95% CI; and (4) review papers, comments, letters or duplicate publications.

When several publications reported on the same TNBC patient group, the most complete publication was included. Moreover, when one publication reported on two or more different independent TNBC patient groups, and the corresponding HR values and 95% CI were provided or could be calculated by Kaplan-Meier curves, the publication was considered as two or more independent studies.

### Definition of oncogenic miRNAs and tumor suppressor miRNAs

According to previous published studies, miRNAs were consideres as tumor suppressive or protective when they were down-regulated compared with normal counterpart, in another word, these miRNAs were associated with an HR value larger than one, otherwise, they were called oncogenic miRNAs or risky miRNAs. In this meta-analysis, each included original study employed different miRNAs, a total HR value was firstly obtained, and then subtotal HR values for oncogenic miRNAs or tumor suppressor miRNAs were calculated.

### Quality assessment and data extraction

Two reviewers separately assessed the quality of the included studies using the guideline of the Newcastle-Ottawa Quality Assessment Scale (NOS) [28], and every study was marked with scores ranging from 0 to 9. The studies with scores greater than a 6 were considered high quality and included in this study. Otherwise, they were removed to enhance the quality of the meta-analysis.

Two reviewers separately extracted the following data from all eligible studies: name of the first author, year of publication, sample number, sample source, type of miRNA(s), methodology, definition of cut-off, follow-up, HR values, 95% CI and P value of miRNAs for predicting overall survival (OS), disease-free survival (DFS), relapse-free survival (PFS), and the distant metastasis-free survival (DMFS). If two HR values were provided by univariate and multivariate analyses, the HR values from the latter were selected as this value considers confounding factors, which should yield more precise predictions. However, when the HR values and corresponding 95% CIs were not directly provided, we calculated these values using appropriate summary statistics or Kaplan-Meier curves as described by Tierney et al [29]. Discrepancies or disagreements were resolved by discussion.

### Statistical analysis

STATA version 12.0 (Stata Corporation, College Station, TX, USA) was adopted to perform all of the meta-analyses. P values were two-sided and P<0.05 was considered statistically significant. All HR values and their corresponding 95% CIs were used as original data to investigate the collected prognostic value of the overall survival or disease-free survival of TNBC. Generally, a worse prognosis of TNBC was indicated by miRNA over-expression, with pooled HR values over 1.00. Heterogeneity among the HR values was assessed by Cochran’s Q test and Higgins’s I2 statistics. Heterogeneity was taken into consideration when P<0.10 and I2>50%, in which case the random-effect model would be used for the meta-analysis. Otherwise, a fixed-effect model would be used to calculate the pooled HR values. Subgroup analyses or a meta-regression was carried out when necessary. Publication bias was determined by Begg’s funnel plot or Egger’s bias test.

## Results

### Literature search and study characteristics

**Fig 1:**
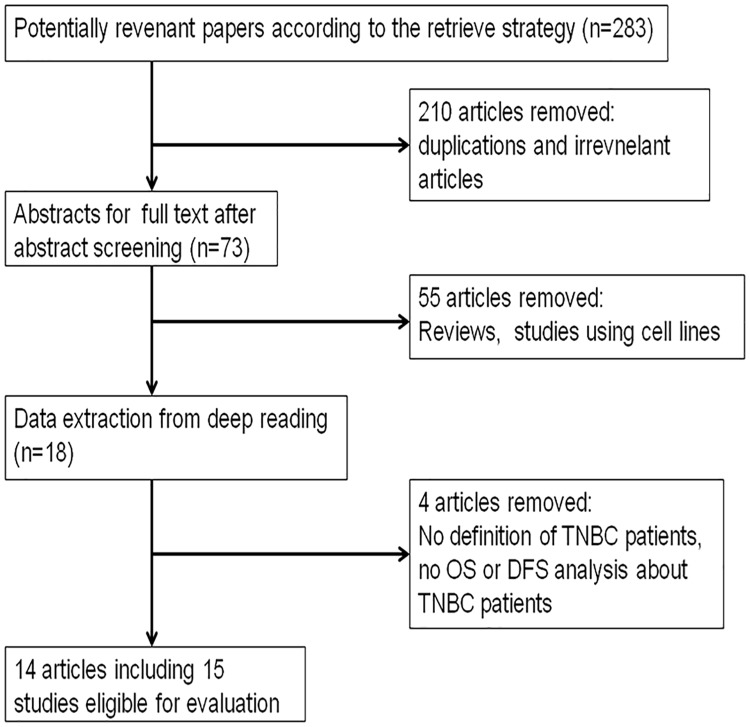
Flow diagram of the identification and selection of the studies.

We initially acquired a total of 283 relevant items from Web of Science, PubMed, EMBASE, and CNKI, according to the retrieval strategy, and subsequently selected the most relevant 73 articles for a full-text review after the abstract screening. Under the guidance of the inclusive and exclusive criteria, 59 articles were removed because they were systematic reviews, basic preclinical studies, or articles lacking the association of OS or DFS with patients with TNBC. Eventually, 14 articles including 15 studies were included in this meta-analysis (Fig 1).

The characteristics of the included studies and the enrolled patients are presented in Tables 1 and 2, respectively. The 15 studies were published between 2012 and 2015, and a total of 1473 TNBC patients from China (n = 7), the USA (n = 3), Norway (n = 2), Japan (n = 2) and Latvia (n = 1) were enrolled in the meta-analysis. Most of the studies detected the expression of miRNA(s) in the tissues by RT-PCR, and the definitions of cut-off for miRNA expression were different from one other. All of the studies analyzed the predictive value of miRNAs of OS in TNBC patients, and 8 studies also analyzed the association of miRNA(s) with DFS, RFS and DMFS in TNBC patients. Furthermore, we evaluated the quality of each individual study and obtained a median NOS score of 8, which indicated that the included studies were of high quality.

**Table 1:** Characteristics of the studies included in this meta-analysis.

Study ID	Country	Patients	Control	Sample	miRNA	Method	Cut-off	Survival analysis	HR	Follow up (m)	Ref
Yu 2014	China	118	118	tissue	miR-301a	RT-PCR	median	OS	Reported	<144	[12]
Cascione 2013	USA	39	36	tissue	miR-125b, miR-497, miR-155, miR-16, miR-374a, miR-421, miR-655, miR-374b,	RT-PCR	>2-fold	OS/DFS	Reported	79 (9–194)	[13]
Dong 2014	China	72	25	tissue	miR-21	RT-PCR	>1.5-fold	OS	Reported	>60	[14]
Gasparin 2014	USA	160	-	tissue	miR-155, miR-493, miR-30e, miR-27a	miRNA microarray	median	OS	Reported	82	[15]
Sahlberg 2014	Norway	40	63	serum	miR-18b, miR-103, miR-107, miR-652	RT-PCR	median	OS/RFS	Reported	>60	[16]
Sahlberg 2014	Norway	70	-	serum	miR-18b, miR-103, miR-107, miR-652	RT-PCR	median	OS/RFS	Reported	>60	[16]
MacKenzie 2014	USA	105	-	tissue	miR-21	ISH	>3	OS	Reported	124	[17]
Shen 2014	China	58	31	tissue	miR-27b-3p	RT-PCR	ROC	OS/DMFS	DE	68 (60–127)	[18]
Toyama 2012	Japan	58	103	tissue	miR-210	RT-PCR	>4	OS/DFS	Reported	64.6 (3–149)	[19]
Kalniete 2015	Latvia	32	18	tissue	miR-214	RT-PCR	median	OS	DE	40	[20]
Liu 2015	China	456	-	tissue	miR-126-3p, miR-374b-5p, miR-218-5p, miR-27b-3p,	miRNA microarray	median	OS/DFS	DE	63.6 (8.4–106.8)	[21]
Liu 2015	China	41	74	tissue	miR-26a	ISH	>3	OS	DE	<120	[22]
Shinden 2015	Japan	68	-	tissue	miR-15a	RT-PCR	median	OS/DFS	DE	<60	[23]
Tang 2014	China	51	51	tissue	miR-185	ISH	>2	OS/DFS	Reported	74	[24]
Yu 2015	China	30	-	tissue	miR-182	RT-PCR	median	OS	Reported	<60	[25]

OS: overall survival; DFS: disease-free survival; DMFS: distant metastasis-free survival; RFS: relapse-free survival, RT-PCR: real-time polymerase chain reaction; DE data extrapolated; ROC: receiver operating characteristic; ISH: in situ hybridization.

**Table 2:** Characteristics of the patients enrolled in the included studies.

Study	age		Grade			Node status			tumor size (cm)			stage				
	<50	>50	1+2	3+4	0	positive	negative	unknown	<2	>2	unknown	1	2	3	4	0
Dong 2014	36.0	36.0	33	39	0	35	37	0	13	59	0	33		39		0
Gasparin 2014	74.0	86.0	15	142	3	58	93	9	-	-	-	-		-		-
Toyama 2012	21.0	37.0	19	39	0	40	17	1	15	43	0	-		-		-
MacKenzie 2014	-	-	-	-	-	-	-	-	-	-	-	-		-		-
Yu 2014	58.0	60.0	104	4	0	47	51	20	74	43	1	84		34		0
Tang 2014	29.0	22.0	30	21	0	18	33	0	27	24						
Shinden 2015	-	-	-	-	-	-	-	-	-	-	-	-		-		-
Liu 2015	-	-	-	-	-	-	-	-	-	-	-	-		-		-
Yu 2015	51.1 (31.0–73.0)		15	15	0	13	17	0	28	2	0	24		6		0
Liu 2015	51.6 (26.1–174.3)		191	263	2	-	-	-	-	-	-	396		47		13
Kalniete 2015	(27.0–78.0)		4	39	7	15	35	0	13	37	0	31		19		0
Sahlberg2014	60.0		14	25	1	18	22	0	-	-	-	27		13		0
Sahlberg 2014	58.0		27	40	3	35	35	0	-	-	-	36		122		0
Cascione2013	43.0 (20.0–50.0)		7	74	5	30	50	6	-	-	-	-		-		-
Shen 2014	46.5 (25.0–79.0)		19	28	11	36	22	0	26	32	0	-		-		-

### Meta-analysis of miRNA(s) in predicting the prognosis of TNBC patients

**Fig 2:**
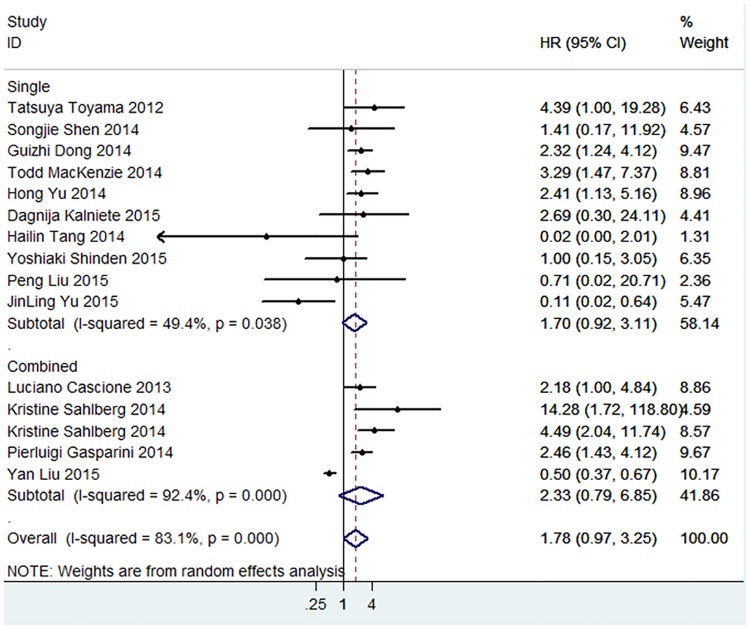
Meta-analysis of subtotal HRs based on single miRNAs and combined miRNAs in predicting the OS of TNBC patients.

**Fig 3:**
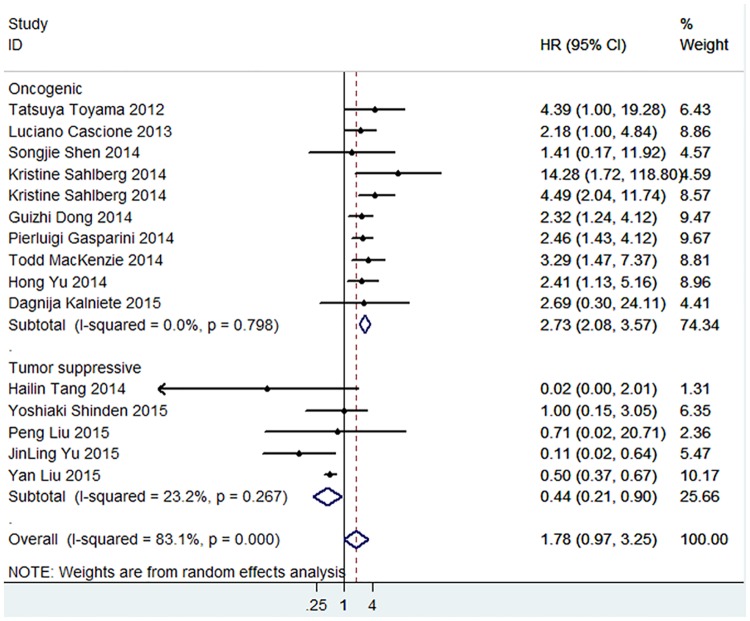
Meta-analysis of subtotal HRs based on oncogenic miRNAs and tumor suppressive miRNAs in predicting the OS of TNBC patients.

A comprehensive meta-analysis was conducted to investigate the predictive value of total miRNAs in the prognosis of TNBC patients, and the pooled HR (1.78, 95% CI: 0.97–3.25) showed a shorter OS of TNBC patients with significant heterogeneity (P<0.001, I^2^ = 83.1%). HR values of single miRNA and combined miRNA subgroups were also obtained, and a worse prognosis was shown for the OS of TNBC patients by the detection of combined miRNAs (Fig 2). More importantly, as miRNAs played distinguished roles in the pathogenesis of TNBC, protective or promotive roles were shown in the studies, and a subgroup analysis was performed within the meta-analysis. Of the 15 studies, 10 employed oncogenic miRNAs and 5 tumor suppressor miRNAs. Subtotal HRs were 2.73 (95% CI: 2.08–3.57; P<0.001) and 0.44 (95% CI: 0.21–0.90; P = 0.024) for oncogenic miRNAs and tumor suppressive miRNAs, respectively, and no heterogeneity was observed in the subgroup analysis (Fig 3).

The same procedure was applied to investigate the pooled HRs for the DFS, RFS and DMFS of TNBC patients, however, we found that However, we then realized that DFS, RFS and DMFS represented different clinical outcomes, it was inappropriate to treat them as equal. And considering that there were too few analogical studies to conclude solid results about DFS, RFS or DMFS, we thought it would be better to not to perform revenant mata-analysis. Subgroups based on patients’ nationality, methodology for miRNA detection, and sample source were also analyzed to investigate the HR values of miRNAs in predicting OS of TNBC patients, as shown in Table 3.

**Table 3:** The results of the subgroup analysis.

Subgroup	N	HR	LL	UL	*P*	*I*^2^	*P* for heterogeneity
Total	15	1.78	0.97	3.25	0.063	83.1%	0.000
Methodology							
RT-PCR	10	2.21	1.29	3.77	0.004	51.5%	0.029
miRNA microarray	2	1.09	0.23	5.21	0.913	96.2%	0.000
ISH	3	0.90	0.08	10.69	0.936	54.8%	0.110
Sample source							
Tissue	13	1.46	0.78	2,73	0.234	82.5%	0.000
Blood	2	5.32	2.37	11.93	0.000	0.00%	0.322
Country							
China	7	0.82	0.32	2.15	0.693	47.0%	0.170
USA	3	2.55	1.74	3.76	0.000	0.00%	0.759
Norway	2	5.32	2.37	11.93	0.000	0.00%	0.322
Japan	2	2.11	0.50	12.78	0.313	47.0%	0.170
Latvia	1	2.69	0.30	24.12	0.377	-	-

N: number of studies; HR: hazard ratio; LL: lower limit of 95% CI; UL: upper limit of 95% CI; RT-PCR: real-time polymerase chain reaction; ISH: in situ hybridization.

### Sensitivity analysis

**Fig 4:**
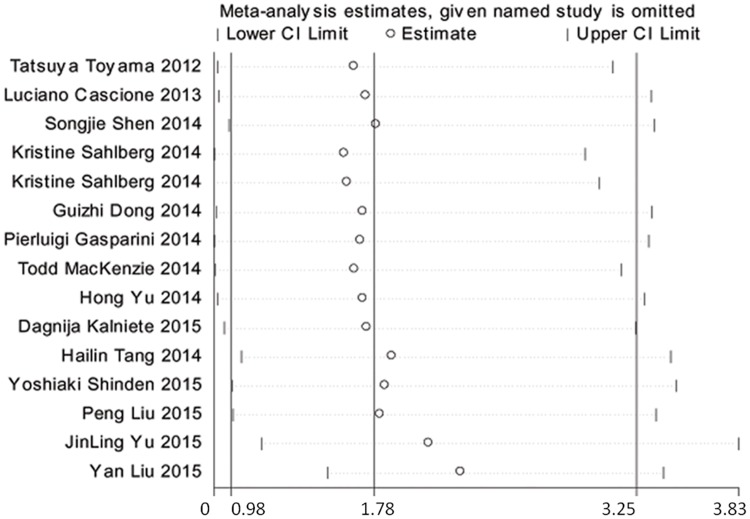
Sensitive analysis of meta-analysis for miRNAs in the prediction of OS.

Although no heterogeneity was observed within the subgroups of oncogenic miRNAs and tumor suppressor miRNAs, significant heterogeneity was shown in the comprehensive meta-analysis. Therefore, a sensitivity analysis was performed to explore the source of the heterogeneity, and the results showed that the re-pooled HRs were not influenced when any specific study was omitted. (Fig 4).

### Publication bias

**Fig 5:**
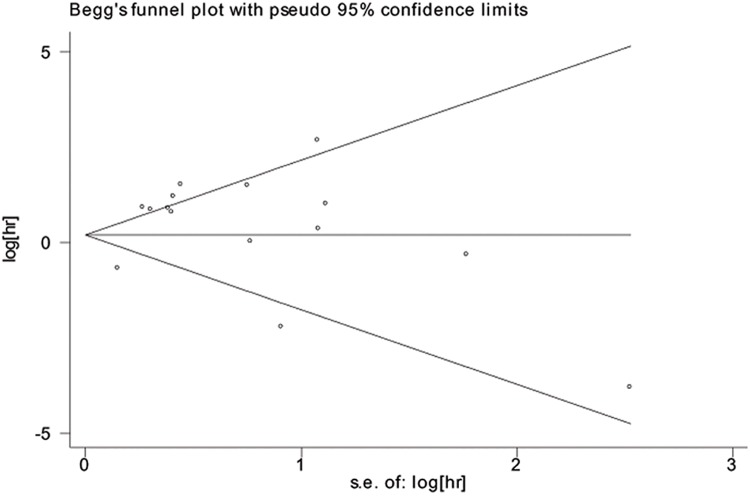
Begg’s funnel plot for publication bias of OS meta-analysis.

Egger’s and Begg’s tests were used to determine whether publication bias existed in the included studies. The Begg’s funnel plots for the OS meta-analysis and non-OS meta-analysis are shown in Fig 5, and the P values were 0.206 and 0.108, respectively, which suggested that no publication bias existed.

## Discussion

TNBC accounts for 12–15% of all breast cancers, and it is the most malignant sub-type with the poorest prognosis. Because of the lack of ER, PgR and HER2 expression, TNBC patients are unlikely to benefit from endocrine therapy or anti-HER2 therapy. For these patients, the only therapeutic options are conventional surgery, chemotherapy or radiotherapy. However, even within the same cohort, TNBC patients may respond differently to the same chemotherapy. Thus, it is of great significance to identify suitable prognostic biomarkers for the specific treatment or timely adaption of treatment for TNBC patients [30].

It is well established that miRNAs play important roles in the initiation, development, metastasis and resistance to treatment of TNBC [31,32]. Tumor suppressive miRNAs mediate the degradation or post-transcriptional inhibition of transcripts encoding oncogenes and can target ZEB1/2, HMGB2, Bcl2 and HIF-1α, which contribute to the increased proliferation, invasion, and EMT and reduce the apoptosis of cancer cells. In contrast, oncogenic miRNAs exert the opposite function. Tumor suppressive miRNAs generally tend to be down-regulated in TNBC cells, while oncogenic miRNAs show the opposite change in expression [10]. miRNAs are more stable than mRNA and DNA, are resistant to harsh environments, and can be extracted without degradation from fresh frozen tissues, paraffin embedded tissues and even blood. Because of these specific features, miRNAs have the potential for extensive clinical use [33–35].

Most of the clinical studies focused on miRNAs as potential prognostic or diagnostic biomarkers in cancer patients, including those in TNBC patients with opposite results. To the best of our knowledge, this is the first meta-analysis to summarize previous studies and analyze the association of miRNAs with the prognosis of TNBC patients. Overall, our results revealed that high miRNA expression was associated with shorter OS of TNBC patients, with HR values over 1.00. Meanwhile, significant heterogeneity was observed in the meta-analysis. The source of this heterogeneity was explored by omitting each single study individually and re-pooling the HRs of the remaining studies. However, our results showed that no specific study influenced the overall HR values. Considering that miRNAs play different roles in the pathogenesis of TNBC, we then divided the studies into subgroups based on oncogenic miRNAs and tumor suppressive miRNAs before performing the meta-analysis. As expected, subtotal HRs of oncogenic and tumor suppressive miRNAs for predicting OS of TNBC patients were 2.73 (95% CI: 2.08–3.57) and 0.44 (95% CI: 0.21–0.90), respectively, with no heterogeneity observed within the subgroups. The pooled HR of tumor suppressive miRNAs was significantly lower than that of oncogenic miRNAs, indicating a better OS for TNBC patients with high expression of tumor suppressive miRNAs. Furthermore, the detection of combined miRNAs showed a worse OS compared to the detection of single miRNAs. Hence, we proposed that the combined detection of miRNAs may serve as a stronger prediction methodPrevious meta-analyses confirmed that circulating miRNAs have a great potential in diagnosing human cancers [35,36], as the expression of circulating miRNAs significantly changes in cancer patients compared to healthy control. Circulating miRNAs may serve as ideal prognostic biomarkers, and the results of this meta-analysis support this hypothesis with a high HR value of 5.32 (95% CI: 2.37–11.93, P<0.0001). However, these results should be interpreted with caution because only two studies conducted by one team were included in this analysis [16]. Furthermore, the results from studies on Chinese TNBC patients showed a better OS when miRNAs were highly expressed, and most of the reports focused on the tumor suppressive miRNAs. Whether the different ethnicities enrolled in this meta-analysis influenced the results deserves further investigation.

Through this meta-analysis, we intended to found reliable biomarkers that could guide the clinical doctors with better adjustment of treatment. Effective treatment was considered when the expressions of oncogenic miRNAs were decreased or tumor suppressive miRNAs were increased. On the contrary, proper adjustment should be made when the oncogenic miRNAs or tumor suppressive miRNAs show no significant change treatment.

Furthermore, recognizing the oncogenic miRNAs or tumor suppressive miRNAs showed great application in finding new methods of treating patients with triple negative breast camer. Take miR-21, an oncogenic miRNA as proved by the original study for example, when the drugs based on anti-miR-21were effectively delivered into triple negative breast cancer model with RNA-nano technology, the tumor size and ability to invasion or migration were significantly impaired.

Although this meta-analysis suggested a prognostic role of miRNAs in predicting the outcome of TNBC patients, several limitations should be taken into consideration. First, some of the studies lacked direct HR values, and we had to calculate them from the given data. Although we followed the procedure recommended by Tierney et al., different HR values and corresponding 95% CIs may have been obtained. Second, the miRNAs employed in these studies were quite different, along with the methodologies for miRNA detection and the definition of the cut-off values, which were potentially strong sources of heterogeneity. Third, the number of enrolled TNBC patients was not large enough to obtain solid results, and some detailed information, such as age, tumor size, tumor grade and stage, were missing. Fourth, few studies analyzed the association of miRNAs with DFS, RFS and DMFS of TNBC patients, so it was hard to raise persuasive results about the DFS, RFS and DMFS of included patients. Finally, only two studies focused on circulating miRNAs. Thus, whether circulating miRNAs could serve as powerful biomarkers with a prognostic value for TNBC patients requires more comprehensive and elaborately designed studies.

## Conclusion

In summary, this study demonstrates that increased expression of tumor suppressive miRNAs predicted favorable outcomes of TNBC patients, and the increased expression of oncogenic miRNAs was associated with negative outcomes. Further, circulating miRNAs could serve as powerful prognostic biomarkers, and this should be verified by targeted studies.

## Supporting Information

S1 PRISMA Checklist S1 PRISMA 2009 checklist. (DOC)
