# Expression of Concern: MiRNAs Predict the Prognosis of Patients with Triple Negative Breast Cancer: A Meta-Analysis

**DOI:** 10.1371/journal.pone.0286445

**Published:** 2023-05-25

**Authors:** The PLOS ONE Editors

After this article was published, similarities were noted between this article [1] and submissions by other research groups which call into question the validity and provenance of the reported results.

In response to queries about these concerns, the authors provided S1 File.

The *PLOS ONE* Editors issue this Expression of Concern to notify readers of the unresolved concerns discussed above, and to provide the file received from the authors.

## Supporting information

S1 File An image of data extracted from the studies used in this article [1]. (TIF)
