# Supplementary figures and images for: Expression of Concern: MiRNAs Predict the Prognosis of Patients with Triple Negative Breast Cancer: A Meta-Analysis
Source: PLoS One. 2023 May 25;18(5):e0286445. doi: 10.1371/journal.pone.0286445 (PMC10212074; doi:10.1371/journal.pone.0286445)

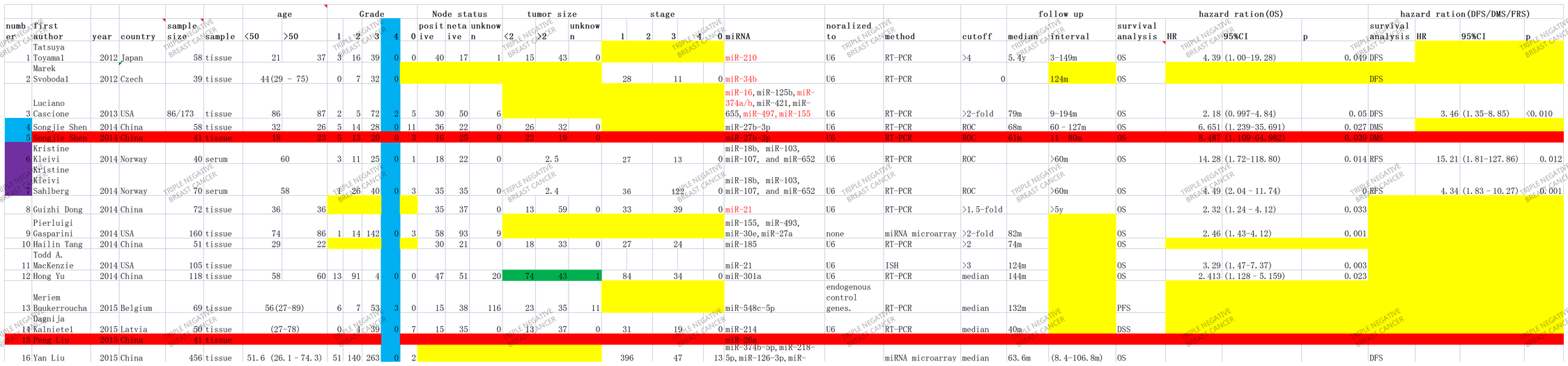

Supplement: S1 File — (TIF) [file pone.0286445.s001.tif]
